# A remote-controlled generation of gold@polydopamine (core@shell) nanoparticles via physical-chemical stimuli of polydopamine/gold composites

**DOI:** 10.1038/srep22650

**Published:** 2016-03-04

**Authors:** Yi Seul Lee, Ji Young Bae, Hye Young Koo, Young Boo Lee, Won San Choi

**Affiliations:** 1 Department of Chemical and Biological Engineering, Hanbat National University, 125 Dongseodaero, Yuseong-gu, Daejeon 305-719, Republic of Korea; 2 Korea Institute of Science and Technology (KIST) Jeonbuk Institute of Advanced Composite Materials, 92 Chudong-ro, Bongdong-eup, Wanju-gun, Jeollabuk-do, Republic of Korea; 3 Jeonju Center, Korea Basic Science Institute (KBSI), Dukjin dong 1-ga, Dukjin-gu, Jeonju 561-756, Republic of Korea

## Abstract

We present the synthesis of polydopamine particle-gold composites (PdopP-Au) and unique release of Au@Pdop core@shell nanoparticles (NPs) from the PdopP-Au upon external stimuli. The PdopP-Au was prepared by controlled synthesis of AuNPs on the Pdop particles. Upon near infrared (NIR) irradiation or NaBH_4_ treatment on the PdopP-Au, the synthesized AuNPs within the PdopPs could be burst-released as a form of Au@Pdop NPs. The PdopP-Au composite showed outstanding photothermal conversion ability under NIR irradiation due to the ultrahigh loading of the AuNPs within the PdopPs, leading to a remote-controlled explosion of the PdopP-Au and rapid formation of numerous Au@Pdop NPs. The release of the Au@Pdop NPs could be instantly stopped or re-started by off or reboot of NIR, respectively. The structure of the released Au@Pdop NPs is suitable for a catalyst or adsorbent, thus we demonstrated that the PdopP-Au composite exhibited excellent and sustained performances for environmental remediation due to its capability of the continuous production of fresh catalysts or adsorbents during the reuse.

Appropriate treatment of pollutants released from our environment is a great challenge. Nanomaterials have been considered as important tools for environmental remediation benefited from their large specific surface areas and high reactivities. The high surface area-to-mass ratio of nanomaterials can remarkably enhance the adsorption or catalytic performance of nanomaterials. However, it is very difficult to prepare nanomaterials of sustained high efficiency because most of nanomaterials suffer from contamination or deformation during repeated reuse^1^,^2^,^3^. Hierarchical nanomaterials have been proposed as feasible materials capable of recycling or maintaining efficiencies, compared to general nanomaterials^4^,^5^,^6^,^7^,^8^,^9^,^10^. Nevertheless, the long-term efficiencies of hierarchical nanomaterials should be further improved, and development of sustained nanomaterials for environmental remediation is highly demanded.

Since the first reports on polydopamine (Pdop) films^11^, Pdop has attracted considerable interest due to the single step formation of Pdop films based on the self-polymerization of dopamine under mild conditions on various substrates regardless of surface characteristics^12^,^13^,^14^,^15^,^16^,^17^,^18^. Pdop can be easily deposited onto various types of organics and inorganics, with controllable film thickness and reliable stability. Thus, Pdop is mainly used as a coating material on various substrates or nanoparticles (NPs). Controllable Pdop shell as a coating layer can be formed on organic or inorganic NPs and the Pdop shell can be further surface-functionalized to improve the NP stability and molecular recognition^12^. Because Pdop possesses biocompatibility and negligible cytotoxicity properties, it can significantly enhance adhesion and proliferation of cells; Pdop coating can thus also be used for tissue engineering^19^,^20^,^21^,^22^. Another valuable feature of Pdop lies in many functional groups such as amine, catechol, and imine, which can serve as both the reaction sites and the anchors for the loading of metal ions^11^,^12^,^13^,^14^. Due to its reducing ability toward various metal ions, Pdop can serve as a good candidate for coating materials as well as templates for preparing nanocomposites. Many previous studies examined the use of Pdop as a template. However, the use of Pdop as a template is restricted to the synthesis and application of NPs on the Pdop^23^,^24^,^25^,^26^. The study for Pdop nanocomposites in a new form and their physicochemical behaviors under external stimuli is highly desirable with much potential for future applications and fundamental studies.

Surface engineering of various NPs is of growing interest and an important step because the surface coating of NPs can protect the core layers from external stimuli, control the surface properties of NPs, and confer them with new functionalities^27^,^28^,^29^. Much effort has been devoted to designing stable surface coatings for NPs using organics or inorganics^30^,^31^,^32^,^33^. General methods for building a surface coating on top of the core NPs include chemical synthesis, conjugation, layer-by-layer assembly, and hydrolysis^34^,^35^,^36^,^37^. For all these methods, NPs are first formed, followed by the formation of a protective shell coating. These types of approaches for core@shell NPs are sometimes time-consuming and involve multiple steps. In terms of the diversity of approaches, significant interest has been shown in developing novel approaches for the preparation of core@shell NPs.

Herein, we report a remote-controlled explosion of the PdopP-Au and rapid formation of numerous Au@Pdop NPs under near infrared (NIR) irradiation. The release of the Au@Pdop NPs can be instantly stopped or re-started by off or reboot of NIR, respectively. The PdopP-Au composite exhibits excellent and sustained performances for environmental remediation due to a continuous supply of fresh nanoadsorbents or nanocatalysts as a form of Au@Pdop NPs by NIR irradiation.

## Results

**Figure 1:**
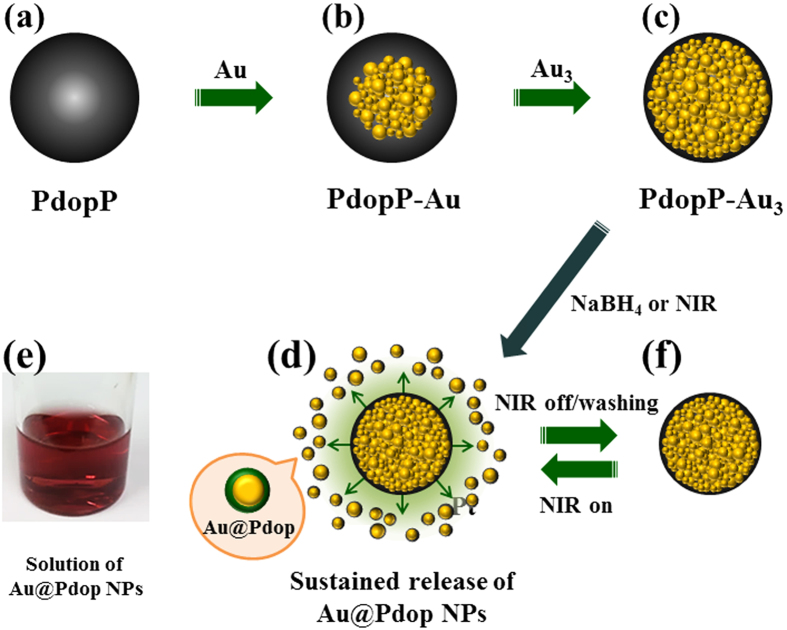
Schematic illustration for synthesis of PdopP-Au composites, producing and releasing Au@Pdop NPs constantly under external stimuli.

Figure 1 shows a schematic illustration of the synthesis of PdopP-Au_3_ composites, which are prepared by stepwise synthesis of AuNPs on the PdopPs (Fig. 1a–c). For the following discussions, we denote the AuNP-synthesized PdopPs as PdopP-Au_n_, where n is the number of repetitions for the AuNP synthesis cycles. The NPs as core@shell (Au@Pdop) structures can be burst released from the PdopP-Au_3_, by chemical treatment or NIR irradiation (Fig. 1d). The released AuNPs have Pdop shell in their surface, and thus they exhibit excellent dispersion stability in the aqueous solution (Fig. 1e). Upon NIR on-off-reboot, release of the Au@Pdop NPs can be instantly started, stopped, or restarted (Fig. 1f). The PdopP-Au composites exhibit excellent and sustainable performances for environmental remediation due to a constant and continuous production of fresh catalysts and adsorbents such as the Au@Pdop (catalyst@adsorbent) NPs during the NIR irradiation.

**Figure 2:**
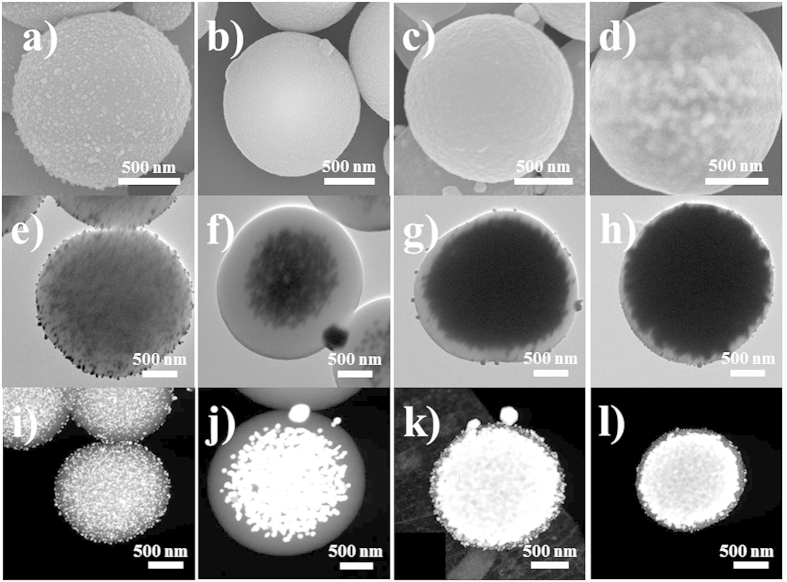
SEM, TEM, and STEM images showing the formation process of the PdopP-Au. SEM, TEM and STEM images of (**a**,**e**,**i**) PdopP-Au(low conc.), (**b**,**f**,**j**) PdopP-Au_1_, (**c**,**g**,**k**) PdopP-Au_2_, and (**d**,**h**,**l**) PdopP-Au_3_.

Figure 2 shows scanning and transmission electron microscopy (SEM and TEM) images of each step for the formation of the PdopP-Au_3_ that were prepared by AuNP synthesis three times within the PdopPs. We found that the synthesized AuNPs can show two different aspects for their occupation within the PdopPs according to the concentration of the Au precursor that participated in the reaction. When the Au precursor of sufficiently low concentration was used, the resulting AuNPs were homogeneously synthesized at the surface of PdopPs (Fig. 2a,e,i). No unusual phenomenon was observed upon repetition of the AuNP synthesis cycles (data not shown). However, in the case of using a high concentration of the Au precursor (Au_1_, Au_2_, and Au_3_), a very interesting phenomenon was observed. The PdopP-Au_1_ and the PdopP-Au_2_ showed a relatively smooth surface morphology (Fig. 2), while the surface morphology of the PdopP-Au_3_ remained unchanged, yet the AuNPs were projected onto the surface of the PdopP-Au_3_ (Fig. 2d). As a common feature of the PdopP-Au synthesized from the high concentration of the Au precursor, corresponding TEM and scanning TEM (STEM) images revealed that an extremely high amount of the AuNPs was unusually concentrated at the center of the PdopPs (Fig. 2f–h,j–l). As the reaction cycle for synthesis of the AuNPs increased, the AuNPs gradually fill the inner space of the PdopPs from center to edge and finally occupy most of the inner space of the PdopPs. These phenomena seem to be quite unusual because generally, metal NPs are apt to precipitate at the surface of templates first and it is not easy to reach the center core of the template even though the metal NPs are synthesized in large amounts.

To investigate this unusual synthetic behavior of the AuNPs within the PdopPs, we conducted control experiments. When we used carbonized PdopPs for the AuNP synthesis, the AuNPs were synthesized only at the surface of the carbonized PdopPs (Figure S1). Catechol and amine groups of the Pdop before carbonization are known as main reaction sites for synthesizing AuNPs^38^. After carbonization of the PdopPs, the amine groups survived, while most of the catechol groups had disappeared^38^. Also, the average particle size of the PdopPs after carbonization decreased to 54% of the original PdopPs of an average size of 1 μm (Figure S2). From these observations, we speculate that diffusion of the Au ions into the carbonized PdopPs might be difficult due to the increased density, compared to the pristine PdopPs that have a relatively low density. Thus, we can conclude that the existence of the catechol groups and the low density of the PdopPs lead to the unusual synthetic behavior of AuNPs within the PdopPs. To obtain further insight, other novel metal NPs such as silver (Ag) and platinum (Pt) were synthesized within the PdopPs under the same experimental conditions. They were synthesized only at the surface of the PdopPs and NPs were not present at the center of the PdopPs (Figure S3), indicating that special interactions between the Au ions and the catechol groups are necessary even if the PdopPs with a low density are used. A previous study supports our results, where it was demonstrated that the catechol groups of PdopPs show highly efficient reactive adsorption and excellent selectivity for Au ions, compared to other metal ions^39^. To summarize the above results, in the case of using a low concentration of Au ions, because of their insufficient amount, most of the Au ions are captured by the catechol and amine groups at the surface of the PdopPs before the Au ions reach the center of the PdopPs. Actually, the yellowish solution color of the Au ions became transparent after reaction, indicating that the functional groups of the PdopPs are still unsaturated by the Au ions (Figure S4). In comparison, for using a high concentration of Au ions, because of the sufficient Au ions capable of intrinsic interaction with the catechol groups, they can reach the functional groups at the surface as well as at the center of the PdopPs by diffusion. Furthermore, a relatively low density of PdopPs also helps the Au ions to easily reach the centre of the PdopP through the pores or channels within the PdopPs. After reaction, the solution color of the Au ions was still yellowish, indicating that the functional groups of the PdopPs were fully saturated by the Au ions (Figure S4). Highly concentrated Au ions accumulated at the PdopP center would be reduced by the functional groups or reducing agents. Therefore, as the reaction proceeded, the PdopPs are gradually filled with the AuNPs from the center to the edge.

**Figure 3:**
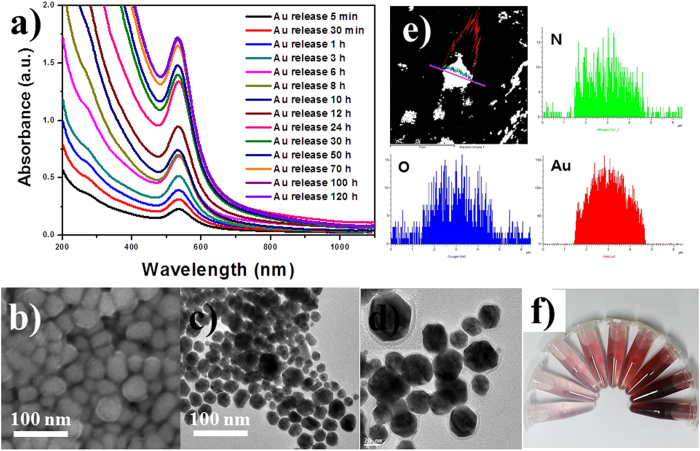
(**a**) UV-vis absorption spectra of the Au@Pdop NPs released from the PdopP-Au_3_ as a function of increasing time. (**b**–**d**) SEM and TEM images of released Au@Pdop NPs. Inset of d shows a magnified image of the released Au@Pdop NPs. (**e**) EDX line scanning data of released Au@Pdop NPs. (**f**) Accumulated solution image of Au@Pdop NPs released as a function of time.

We observed another very interesting phenomenon as a result of the treatment of NaBH_4_ on the PdopP-Au_3_. When the resulting PdopP-Au_3_ was dispersed in water after treatment of NaBH_4_ (150 mM), a red colored solution slowly appeared from the PdopP-Au_3_. Figure 3a shows the time-dependent UV-vis spectra of the red solution released from the PdopP-Au_3_. A strong absorbance peak near 530 nm appeared due to the surface plasmon resonance (SPR) of AuNPs, confirming the release of the AuNPs from PdopP-Au_3_. The intensity of the absorbance gradually increased over a period of 120 hours as the reaction proceeded, indicating the sustained release of the AuNPs from the PdopP-Au_3_. The PdopP-Au_3_ initially displayed a burst-release of the AuNPs, releasing 14% of the total released amount within 5 minutes (black line), and the release was almost completed after 100 h. Fig. 3b,c show the representative SEM and TEM images of the AuNPs released from the PdopP-Au_3_. The size of the released AuNPs was approximately 5–45 nm. The magnified TEM image clearly shows the AuNPs wrapped with an organic shell with an average thickness of 5 nm, revealing the core@shell structures (Fig. 3d). STEM/EDX data of the released AuNPs confirmed the existence of Au, N, and O peaks, indicating the AuNPs coated with Pdop (Fig. 3e). The presence of the Pdop on the surface of the AuNPs was also confirmed by X-ray photoelectron spectroscopy (XPS) (Figure S5). The released Pdop-coated AuNPs are denoted as the Au@Pdop NPs. It was possible to concentrate the Au@Pdop NPs up to 5.1 wt% without any precipitation, and the Au@Pdop NPs showed excellent dispersibility in aqueous solution for a period of at least 3 months, due to the very thin and charged shell of Pdop (Fig. 3f). After the release of Au@Pdop NPs for 120 hours, the PdopP-Au_3_ decreased in size, and collapse of the sphere structure was observed on the surface of the PdopP-Au_3_ (Figure S6). The release of Au@Pdop NPs resulted in a less plasmon-coupled phenomenon of the Pdop-Au_3_, exhibiting not only the decreased absorbance but also the blue-shifted peak position of the Pdop-Au_3_ (Figure S7).

To investigate the release mechanism of the Au@Pdop NPs, several comparative experiments were performed. To determine whether other types of metal NPs can be similarly released from the PdopPs, the PdopP-Ag_3_ and the PdopP-Pt_3_ were prepared and tested under the same conditions. We could not find any structural collapse of the PdopP-Ag_3_ or the PdopP-Pt_3_ after the release test (Figure S8), and no remarkable SPR peaks of the AgNPs or PtNPs were detected, suggesting that only AuNPs can be released from the Pdops. Also, the Au@Pdop NPs released from the PdopP-Au_1_ and -Au_2_ were not observed. The synthesis of AuNPs over 3 reaction cycles was necessary for the successful release of the Au@Pdop NPs. These results suggest that quite a large amount of the AuNPs possessing selective affinity with catechol groups is necessary for the release of the Au@Pdop NPs. Furthermore, once the PdopP-Au_3_ was carbonized, the Au@Pdop NPs were no longer released from the carbonized PdopP-Au_3_, indicating that the presence of the catechol groups is crucial for the release of NPs because most catechol groups are removed after carbonization^38^. The formation mechanism of Pdop is generally considered to be the result of the combination of covalent interaction by polymerization and non-covalent interactions including hydrogen bonding^12^. In parallel with this model, Pdop is also considered to be an aggregate of monomers that are cross-linked primarily via non-covalent forces including hydrogen bonding, charge transfer, and π-stacking^12^. Thus, hydrogen bonding by catechol groups is one of the important interactions for the formation of Pdop^12^. However, it is expected that a certain portion of hydrogen bonding between the Pdop interchains can be dissociated after synthesis of the AuNPs because catechol groups are one of main reaction sites for synthesizing AuNPs. Upon dissolution of NaBH_4_ in water, the NaBH_4_ reacts with water to produce hydrogen gas and forms a basic condition^40^ (the pH of NaBH_4_ solution was 9.8 for 150 mM). Treatment of NaOH (pH = 9.8) does not result in release of the Au@Pdop NPs from the PdopP-Au_3_ (Figure S9), suggesting that the pH condition caused by the NaBH_4_ has little influence on release of the Au@Pdop NPs. We speculated that hydrogen gases produced by the NaBH_4_ play a role on release of the Au@Pdop NPs. Vibrational motion of hydrogen gases can stimulate the Pdop chains dissociated by synthesis of a large amount of the AuNPs and cause detachment of the Pdop chains as a form of Au@Pdop NPs. This hypothesis can be further supported by results showing release of the Au@Pdop NPs caused by enhanced chain mobility of the Pdop through heat treatment, which will be further discussed later. To sum up, as the amount of the AuNPs synthesized within the PdopPs increases, dissociation of hydrogen bonding further proceeds with asistance of hydrogen gases, leading to the breakage of non-covalent bonding between the Pdop interchains and the release of the Pdop fragments as a form of Au@Pdop NPs.

**Figure 4:**
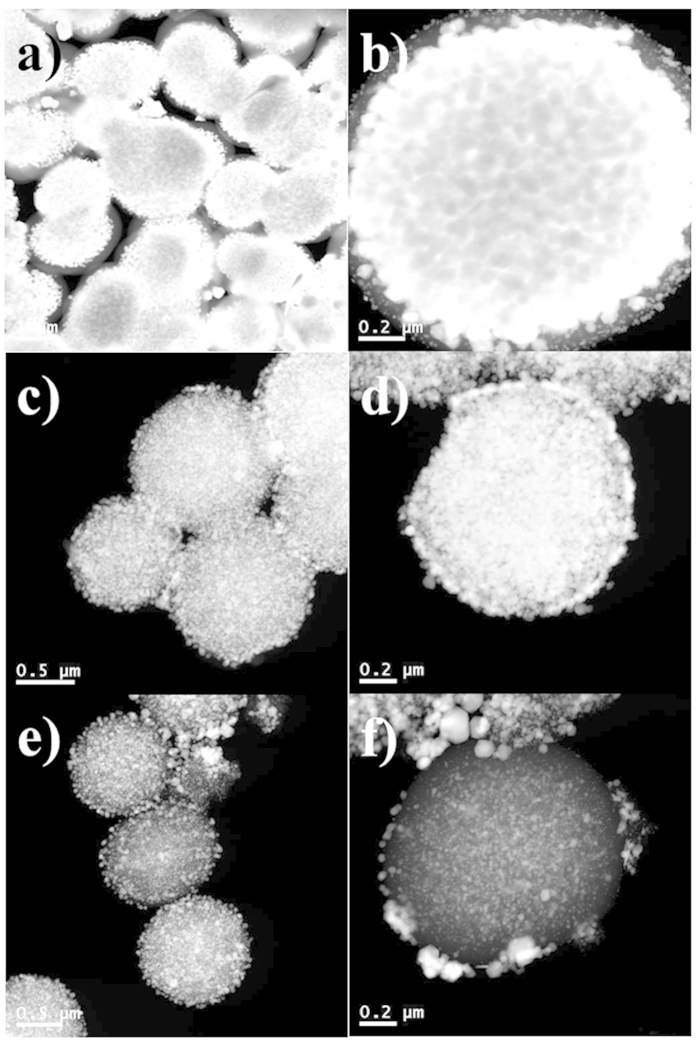
STEM images of PdopP-Au_3_ before and after release of the Au@Pdop NPs. (**a**,**b**) PdopP-Au_3_ before release of NPs. PdopP-Au_3_ after release of NPs for (**c**,**d**) 5 minutes and (**e**,**f**) 100 hours.

To investigate the release pattern of the Au@Pdop NPs, the release process was monitored by STEM. Figure 4 shows the internal structure of the PdopP-Au_3_ before and after the release of the Au@Pdop NPs. Before release, the PdopPs were almost filled with an extremely large amount of the AuNPs, and a relatively low amount of AuNPs was observed at the surface of the PdopP-Au_3_ (Fig. 4a,b). After release of the Au@Pdop NPs for 5 minutes, the electron density of the PdopP-Au_3_ fairly decreased, compared to that of the PdopP-Au_3_ before release, demonstrating the release of the Au@Pdop NPs from the PdopP-Au_3_ (Fig. 4c,d). It is significant that the AuNPs with relatively large sizes migrated to the edge of the PdopP-Au_3_ during the release, which was not observed in the PdopP-Au_3_ before release (Fig. 4b,d), suggesting the release of relatively large sized AuNPs at the beginning of the release. After 100 hours, the amount of AuNPs within the PdopPs decreased significantly and the AuNPs with relatively small sizes remained (Fig. 4e,f).

**Figure 5:**
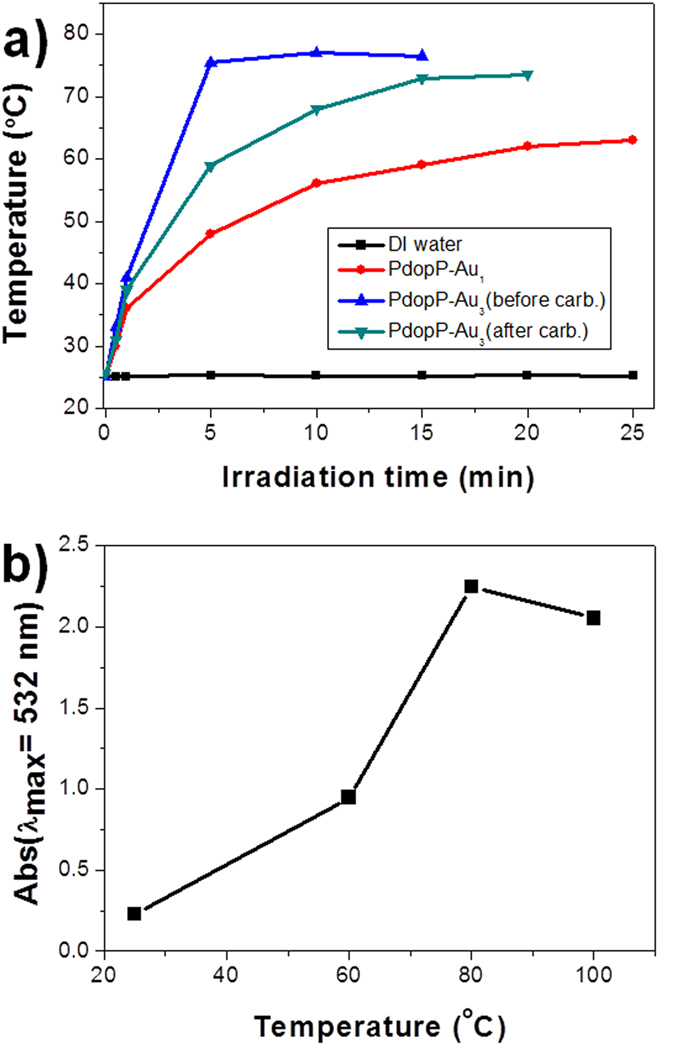
(**a**) Temperature rise traces of the three different PdopP-metal composites at the same concentrations under NIR illumination. (**b**) Maximum absorbance (at 532 nm) of Au@Pdop NPs released from the PdopP-Au_3_ for 5 minutes as a function of increasing temperature.

We investigated the photothermal conversion performances of three different PdopP-metal composites (PdopP-Au_3_ before and after carbonization, and PdopP-Au) at the same concentration. The temperatures of all samples show an increase under near infrared (NIR) irradiation (Fig. 5a). The highest temperatures for the PdopP-Au_3_ before and after carbonization were 77 °C and 73.5 °C, respectively (blue and blush green lines). The solutions of the PdopP-Au_3_ before carbonization reached their steady state temperatures within 5 min, which was considerably faster than that of the PdopP-Au_3_ after carbonization. Even though a small amount (1 mg/10 ml) of the PdopP-Au_3_ was used for the photothermal conversion test, the PdopP-Au_3_ before carbonization exhibited the highest steady state temperature. Another notable feature observed for the PdopP-Au_3_ is that the Au@Pdop NPs can be released from the PdopP-Au_3_ by NIR irradiation. After 3 minutes of NIR irradiation on the PdopP-Au_3_, a red-colored solution containing Au@Pdop NPs was observed with the naked eye, and the solution temperature dramatically increased (Figure S10). Thus, we speculated that the rapid increase in the solution temperature of the PdopP-Au_3_ during initial 5 minutes could be attributed to the release of the Au@Pdop NPs. In contrast, once the PdopP-Au_3_ was carbonized, the Au@Pdop NPs were no longer released from the carbonized PdopP-Au_3_ even under NIR irradiation (Figure S11). To reveal the reason for the release of NPs by NIR irradiation, the following experiments were performed. The influence of temperature on the release of the Au@Pdop NPs was investigated because the solution temperature of the PdopP-Au_3_ increased by up to approximately 80 °C during the NIR irradiation. Figure 5b shows the amount of NPs released from the PdopP-Au_3_ for 5 minutes as a function of temperature. The absorbance at 532 nm was considered to be the released amount of NPs due to the SPR peak of AuNPs. As the temperature increased, the amounts of released NPs remarkably increased and the release speed was much faster than that obtained at room temperature. Maximum release at 80 °C was observed, which is similar to the temperature increased by NIR irradiation on the PdopP-Au_3_. Pdop can endure temperatures up to 200 °C without suffering any significant decomposition^12^. Moreover, since the integrity of the PdopP-Au_3_ before and after NIR irradiation remained unchanged (Figure S12), we believe that the mobility of the Pdop chains enhanced by thermal energy produced through the NIR irradiation triggered a burst release of the Au@Pdop NPs in a short period of time. Upon NIR off or reboot, release of the Au@Pdop NPs could be instantly stopped or restarted. These results suggest that the remote-controlled release behaviors of the Au@Pdop NP can be controlled by NIR irradiation on-off-reboot cycle on the PdopP-Au_3_.

**Figure 6:**
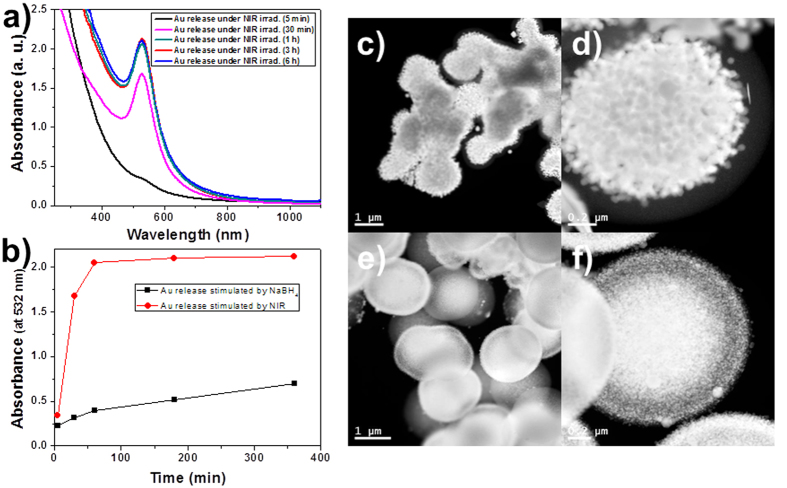
(**a**) UV-vis absorption spectra of the Au@Pdop NPs released from the PdopP-Au_3_ as a function of increasing NIR irradiation time. (**b**) Release efficiencies of the Au@Pdop NPs stimulated by the NaBH_4_ or the NIR. The absorbances at 532 nm were considered to be the released amount of NPs. STEM images of the PdopP-Au_3_ (**c**,**d**) before and (**e**,**f**) after release of the Au@Pdop NPs triggered by the NIR irradiation for 30 minutes.

Release efficiencies of the Au@Pdop NPs stimulated by the NaBH_4_ or the NIR were compared. Figure 6a shows the time-dependent UV-vis spectra of the red solution released from the PdopP-Au_3_ as a function of NIR irradiation time. The intensity of the absorbance suddenly increased after 30 minutes of NIR irradiation, releasing 79% of the total released amount within 30 minutes, and the release was almost completed after 1 h (Fig. 6a,b). For the release case stimulated by the NaBH_4_, less than 20% of the total amount was released in the same period of time, suggesting that the NIR irradiation method is more effective for release of NPs than the NaBH_4_ method (Fig. 6b). Figures 6c–f show the internal structure of the PdopP-Au_3_ before and after the release of the Au@Pdop NPs stimulated by NIR irradiation. After release of the Au@Pdop NPs for 30 minutes, the electron density of the PdopP-Au_3_ fairly decreased, and the AuNPs migrated to the edge of the PdopP-Au_3_ during the release, which were not observed in the PdopP-Au_3_ before release. After NIR irradiation for 6 hours, the release of NPs completed and the size of the PdopP-Au_3_ decreased to 66% of the original size of the PdopP-Au_3_ (Figure S13).

**Figure 7:**
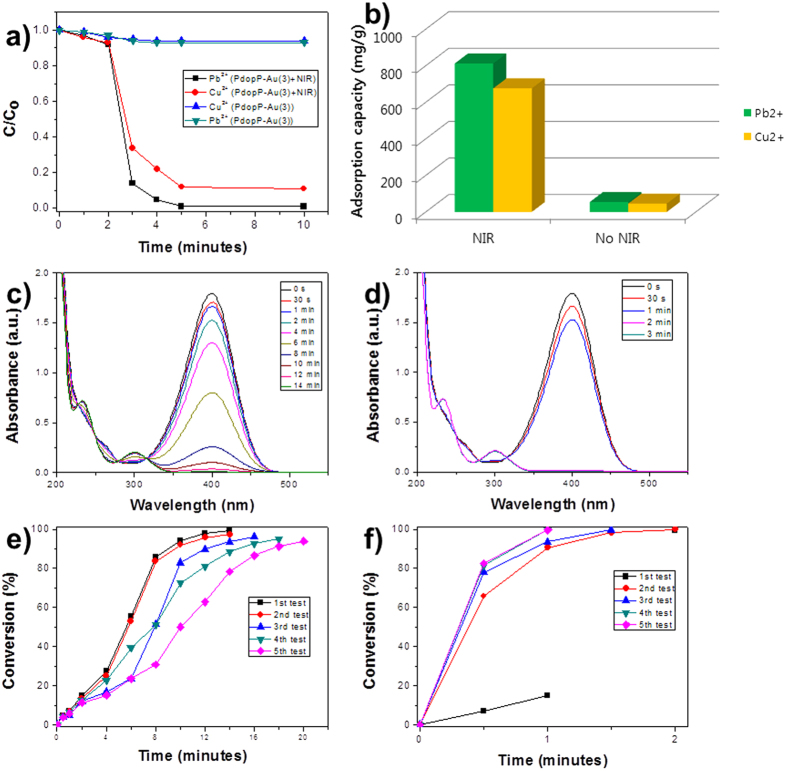
(**a**) Removal efficiencies of Pb^2+^ and Cu^2+^ with PdopP-Au_3_ in the absence and presence of NIR irradiation. (**b**) Comparison of adsorption capacities of Pb^2+^ and Cu^2+^ on PdopP-Au_3_ in the absence and presence of NIR irradiation. (**c**,**e**) Time-dependent UV-vis absorption spectral changes of 4-NPh reduction and (**d**,**f**) their conversion rates in the presence of PdopP-Au_3_ (**c**,**d**) without or (**e**,**f**) with NIR irradiation.

To explore potential applications of the PdopP-Au composite for environmental remediation, the PdopP-Au_3_ was employed as adsorbent for heavy metal ions because the Pdop can be used for adsorption of heavy metal ions^12^. Lead (Pb^2+^) and copper (Cu^2+^) ions were employed for the removal test. Figure 7a shows removal efficiencies of the heavy metal ions when the PdopP-Au_3_ was used as the adsorbent. Before irradiation of NIR, the maximum removal efficiencies of the PdopP-Au_3_ were only 7% for Pb and 6% for Cu by 5 minutes of incubation time. Under irradiation of NIR, however, the removal efficiencies greatly increased as 99% for Pb and as 89% for Cu within 3 minutes and they reached to saturated values after 5 minutes. Results for removal capacity of Pb and Cu were 812 mg/g and 676 mg/g, respectively, which were approximately 15 times increased compared to the PdopP-Au_3_ before NIR irradiation (Fig. 7b). These values are significantly higher than those previously reported systems^8^,^9^. It can be attributed to remarkable increase in the surface area of adsorbents such as Au@Pdop NPs released from the PdopP-Au_3_ upon NIR irradiation. Since the PdopP-Au composite possesses AuNPs suitable for a catalyst, the catalytic activity of the PdopP-Au_3_ was tested. As a model material, the reduction of 4-nitrophenol (4-NPh), which is a toxic material generated from industrial wastewater, to 4-aminophenol (4-APh) was chosen. After addition of the PdopP-Au_3_ into a 4-NPh solution, the conversion rates of the reaction were monitored using Uv-vis absorption spectroscopy. As the reaction progressed, the absorption peak of the 4-NPh at 400 nm diminished with the simultaneous appearance of a new peak at approximately 300 nm, which indicates the formation of the 4-APh (Fig. 7c). The conversion rate after 14 minutes for the reduction of 4-NPh to 4-APh was 99.1%, which was gradually decreased to 93.1% upon reuse test of 5^th^ cycles (Fig. 7d). The decay of catalytic activity is typically observed for general catalytic reuse test. In comparison, the catalytic performance of the PdopP-Au_3_ under NIR irradiation was excellent. The absorption peak of the 4-NPh at 400 nm quickly decreased and reached almost to a zero level only after 2 minutes of incubation time (Fig. 7e). The conversion rate after 2 minutes was 99.8%, and this high conversion rates could be maintained as around 99.6% upon reuse test of 5^th^ cycles (Fig. 7f). Slightly delayed release of the Au@Pdop NPs was observed upon 1^st^ irradiation of the NIR (Fig. 7f, black line), which is attributed to the travel time of the NPs to reach the edge of the PdopP as shown in the Fig. 6d. No deterioration on the catalytic activity suggests that considerable amount of the Au@Pdop NPs are constantly released from the PdopP-Au_3_ under NIR irradiation during the reuse. These results suggest that the NIR irradiation triggers a remote controlled explosion of the PdopP-Au_3_, and the Au@Pdop NPs released from the PdopP-Au_3_ can enhance and maintain adsorption and catalytic performances.

**Figure 8:**
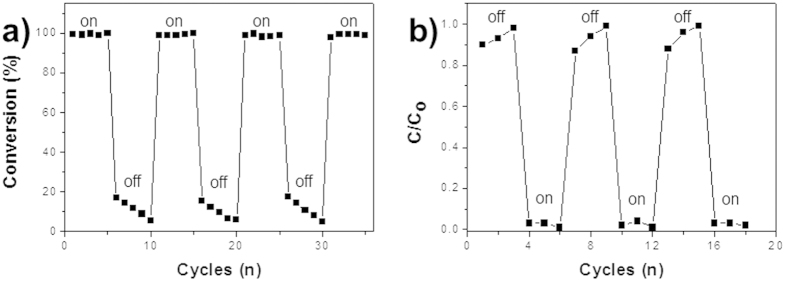
(**a**) Conversion rates of 4-NPh using PdopP-Au_3_ under NIR irradiation on-off-reboot cycle upon reuse. NIR irradiated on and off during each 5 cycle. (**b**) Removal efficiencies of Pb^2+^ with PdopP-Au_3_ under NIR irradiation on-off-reboot cycle upon reuse. NIR irradiated on and off during each 3 cycle.

Sustained performances of the PdopP-Au composite through continuous production of the Au@Pdop NPs was demonstrated by repetition of NIR on and off. Figure 8a shows the conversion rate of the 4-NPh for 2 minutes using the PdopP-Au_3_ as catalyst for reuse reaction. After completion of the first reaction, the PdopP-Au_3_ was separated from the solution, redispersed in the fresh 4-NPh solution, and irradiated by NIR for next reaction. After five consecutive reaction under NIR irradiation, the PdopP-Au_3_ was separated and redispersed in the fresh 4-NPh solution for next reaction under no NIR irradiation. Consecutive five times of NIR irradiation and five times of NIR off was repeated until the desired cycles were obtained. Under the NIR irradiation, high conversion rates were repeatedly observed for every reuse reaction. However, under NIR irradiation off, low and gradually decreased conversion rates were observed, which is commonly observable phenomena for general catalyst during the reuse. It is worthy of mentioning that the concentration of the NaBH_4_ used for the catalytic test was 100 mM. Because for release of the Au@Pdop NPs at least 150 mM of NaBH_4_ concentration is needed, the NaBH_4_ itself used for catalytic test with NIR irradiation does not induce release of the Au@Pdop NPs and thus it does not interfere with the NIR-triggered release of NPs. This could be further confirmed by the result showing the decreased catalytic performance upon NIR off as shown in the Fig. 8a. In this study, NIR reboot restored the conversion rate to original performance of on mode due to fresh release of the Au@Pdop NPs. Similar tendency was also observed in removal test of heavy metal ions. Under the NIR irradiation, high removal efficiencies of the Pb ions by 5 minutes were repeatedly observed for every reuse reaction, compared to a decaying efficiencies under NIR irradiation off (Fig. 8b). On the basis of the UV-vis absorbance ratio of the total released amount of the NPs, approximately 21% of Au@Pdop NPs calculated to be remained within the PdopP-Au_3_ after 30 cycles because the NIR irradiation for 30 min is necessary for 30 cycles in catalytic or adsorption reaction (Fig. 6a). Our material described here showed a sustained performance without deterioration upon reuse, which is not observed in general catalysts or adsorbents. It can be attributed to a continuous production of the fresh Au@Pdop NPs from the PdopP-Au composite by NIR irradiation during the reuse. Even though our approach cannot produce exactly controlled core size or even thickness of the shell, the advantage of our approach for generation of the Au@PdoP as catalyst or adsorbent lies in the point that the fresh catalysts or adsorbents can be generated by simple NIR irradiation for extended performance for the catalytic or adsorption activity, which seems quite novel compared to the previous approaches for production of core@shell particles.

## Conclusions

In conclusion, we demonstrated sustained generation of the Au@Pdop NPs from the PdopP-Au composite with controlled releasing properties. The synthesized AuNPs could be released as core@shell Au@Pdop structures by external stimuli such as chemical treatment or NIR irradiation. The release speed and amount of the Au@Pdop NPs can also be tuned by releasing parameters involving NIR or temperature. The PdopP-Au composite showed outstanding photothermal conversion ability under NIR illumination due to the ultrahigh loading of the AuNPs within the PdopPs, leading to a remote-controlled explosion of the PdopP-Au_3_ and rapid formation of Au@Pdop NPs. Upon NIR off or reboot, release of the Au@Pdop NPs could be instantly stopped or restarted. Along with a NIR irradiation, the PdopP-Au_3_ composite exhibited excellent and sustainable performances for environmental remediation due to a constant and continuous production of fresh Au@Pdop NPs during the reuse. We believe that due to the the remote-controlled release behaviors of NPs, our approach described here could offer significant technological promise in the fields of environmental remediation and core@shell NP synthesis.

## Methods

### Materials

Dopamine hydrochloride, ethanol, ammonium hydroxide solution (NH_4_OH), gold(III) chloride hydrate (HAuCl_4_), chloroplatinic acid hydrate (H_2_PtCl_6_), silver nitrate (AgNO_3_), lead(II) nitrate (Pb(NO_3_))_2_, 99.99%), copper(II) nitrate (Cu(NO_3_))_2_, and sodium borohydride (NaBH_4_) were purchased from Aldrich and used as received. Deionized (DI) water with a resistance of 18.2 MΩ cm was obtained using a Millipore Simplicity 185 system.

### Synthesis of PdopPs

An ammonia solution (NH_4_OH, 0.4 mL) was added to a mixture solution of EtOH (40 mL) and D.I. water (90 mL) under mild stirring at room temperature for 1 hour. An aqueous solution of dopamine hydrochloride (0.5 g/10 mL) was slowly injected dropwise into the above-mentioned mixture solution. The resulting solution was reacted for 30 hours. The products were obtained by centrifugation and rinsed three times with D.I. water.

### Synthesis of PdopP-metal NPs

For PdopP-Au_3_, an aqueous solution of HAuCl_4_ (10 mM) was added to a suspension of the resulting PdopPs. For PdopP-Ag_3_ and PdopP-Pt_3_, an aqueous solution of H_2_PtCl_6_ (10 mM) and AgNO_3_ (10 mM) was used. The dispersion was agitated vigorously on a shaking apparatus for 4 h to allow adsorption of the Au ions on the PdopPs. The rinsing step was repeated three times. The AuNPs were synthesized within the PdopPs by treatment with a NaBH_4_ (5 mM) solution for 30 minutes. The rinsing step was repeated three times with water. The above reaction was repeated until the desired reaction cycle was achieved.

### Photothermal conversion test

The PdopP-metal NPs (Au_3_, Au, 1 mg) were added to a D.I. water (10 mL). The solution was stirred at room temperature. The solution was then exposed to a 1 W NIR laser (800 nm, PSU-III-FDA, Changchun industries) for a period of time and the variations of solution temperature were then recorded. The distance between the solution and the NIR laser was maintained at 0.5 meter.

### Adsorption test of heavy metal ions

Aqueous solutions of Pb(NO_3_)_2_ and Cu(NO_3_)_2_ were used as source materials for Pb(II) and Cu(II), respectively. The initial concentrations of Pb(II) and Cu(II) solutions were 81.2 and 79.8 mg/L, respectively. The PdopP-Au_3_ adsorbents (0.002 g) were added to the metal ion solutions (20 mL) with vigorous stirring. After a specified incubation time with or without NIR irradiation, the adsorbents were separated from the solutions using ultracentrifugation. Inductively coupled plasma mass spectroscopy was used to measure the amount of lead or copper ions remaining in the solution. The metal ion adsorption capacity was calculated by using Equation (1) in which q_e_ is the equilibrium concentration of metal ions on the adsorbent (in mg/g), C_o_ is the initial concentration of the metal solution (in mg/L), C_e_ is the equilibrium concentration of the metal solution (in mg/L), m is the mass of absorbent (in g), and V is the volume of the metal solution (in L)
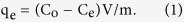


### Catalytic test for reduction of 4-NPh

To investigate the catalytic reduction of 4-nitropheol to 4-AP, 1 mL of water was placed in a quartz cell. 15 mL of freshly prepared 4-NP aqueous solution (0.08 mM) and 700 μL of NaBH_4_ aqueous solution (100 mM) were mixed. 2 mg of PdopP-Au_3_ was added with gentle shaking. After the addition of the PdopP-Au_3_, UV-vis spectra of the mixture were monitored at regular intervals to observe the reaction progress.

### Characterization

The fabricated PdopPs and PdopP-metal NPs (Au_3_, Ag_3_, and Pt_3_) were characterized by field-emission transmission electron microscopy (FE-TEM) using a JEOL JEM 2100F, and a Hitachi S-5500 was used for field-emission scanning electron microscopy (FESEM/EDX) analysis. FT-IR spectra were recorded using a Fourier transform infrared spectrometer (Sinco Nicolet IS5). The UV-vis absorption spectra were recorded on a UV-vis spectrophotometer (Scinco Evolution201). Particle size measurements were performed on a particle size analyzer (Shimadzu, SALD-7500nano). XPS studies were performed on an Axis NOVA (Kratos analytical) spectrometer using an aluminum anode (Al Kα, 1486.6 eV) operated at 600 W.

## Additional Information

**How to cite this article**: Lee, Y. S. *et al.* A remote-controlled generation of gold@polydopamine (core@shell) nanoparticles via physical-chemical stimuli of polydopamine/gold composites. *Sci. Rep.* **6**, 22650; doi: 10.1038/srep22650 (2016).

## Supplementary Material

Supplementary Information
