# Supplementary material for: A remote-controlled generation of gold@polydopamine (core@shell) nanoparticles via physical-chemical stimuli of polydopamine/gold composites
Source: Sci Rep. 2016 Mar 4;6:22650. doi: 10.1038/srep22650 (PMC4778136; doi:10.1038/srep22650)
Supplement: Supplementary Information [file srep22650-s1.pdf]

Supplementary Information

A remote-controlled generation of gold@polydopamine (core@shell) nanoparticles via physical-chemical stimuli of polydopamine/gold composites

Yi Seul Lee,[§,1] *Ji Young Bae,*[§,1] *Hye Young Koo,**[2] *Young Boo Lee,*[3] *and Won San Choi**[1]

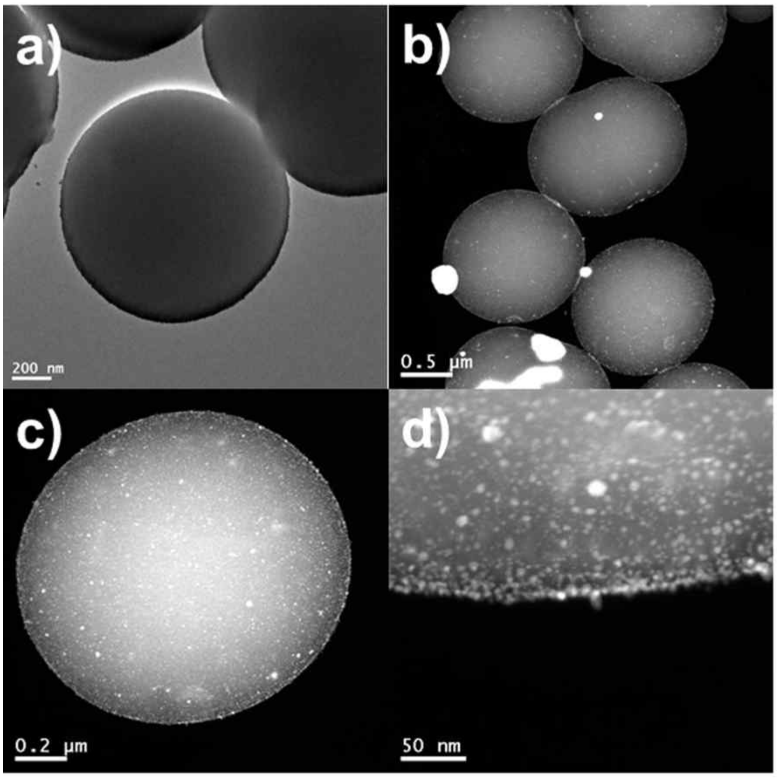

Fig. S1 (a) TEM and (b-d) STEM images of carbonized PdopPs after synthesis of AuNPs (carbonized PdopP-Au_3). The AuNPs were observed only at the surface of carbonized PdopPs.

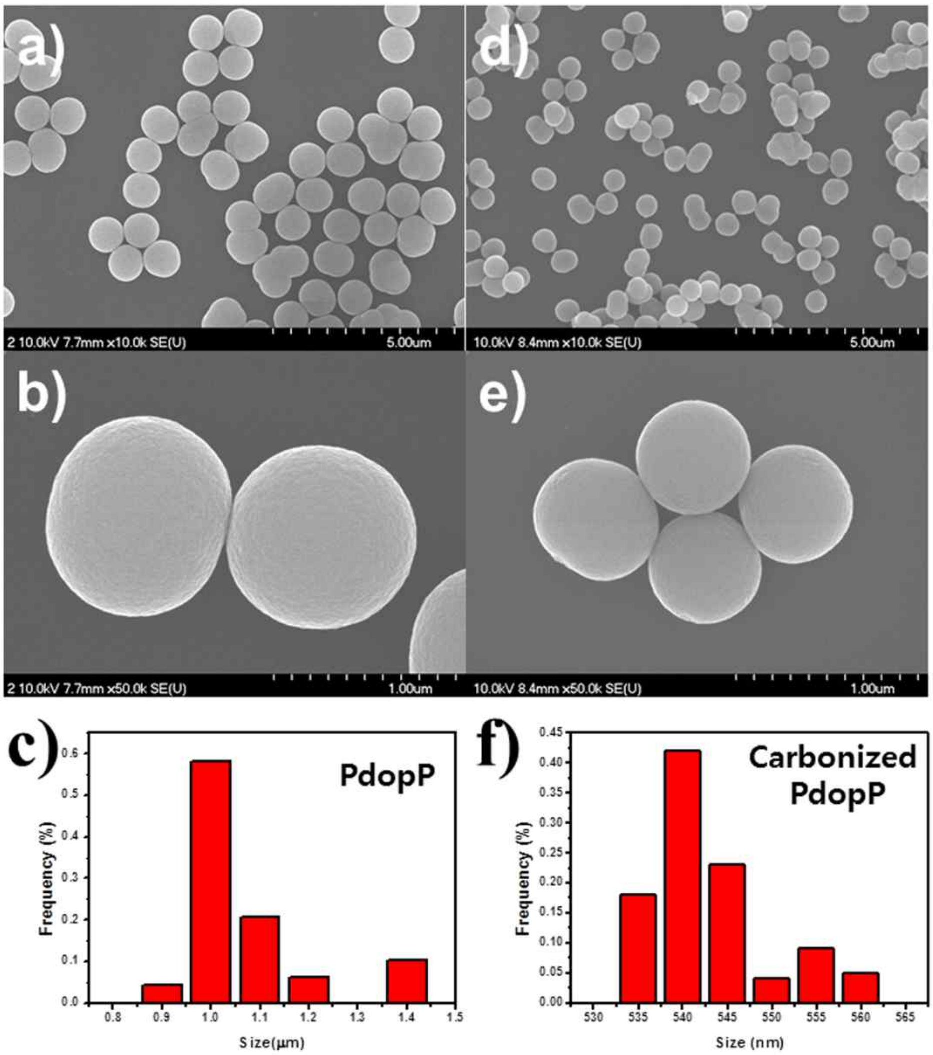

Fig. S2 SEM images and their corresponding size data of PdopP (a-c) before and (d-f) after carbonization.

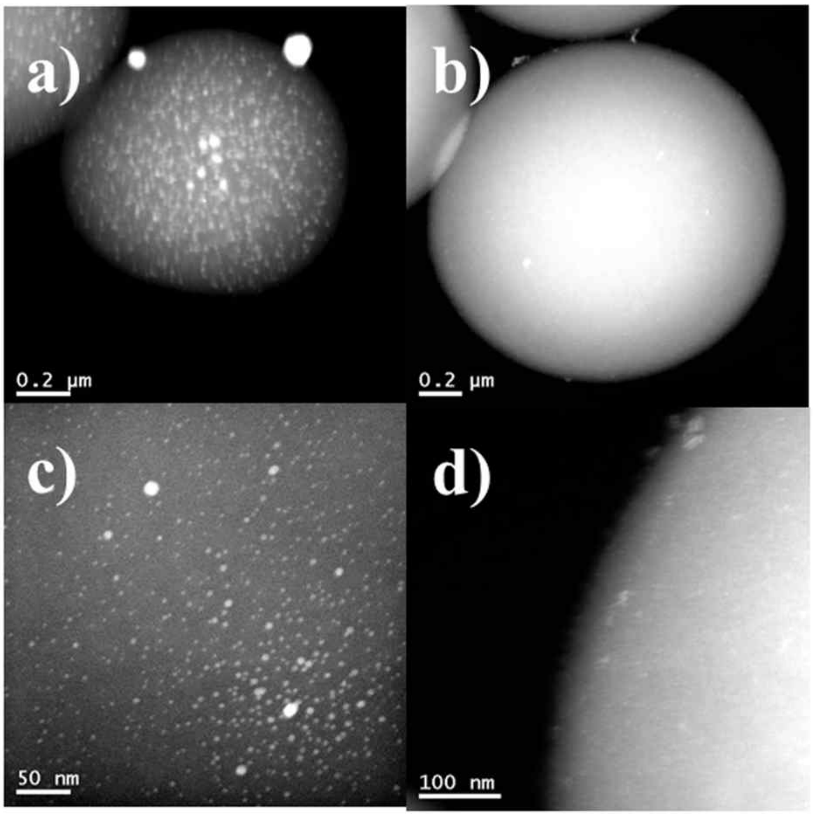

Fig. S3 STEM images of PdopPs after synthesis of (a, c) AgNPs and (b, d) PtNPs. Both of NPs were synthesized only at the surface of the PdopPs and there was no presence of the NPs at the center of the PdopPs.

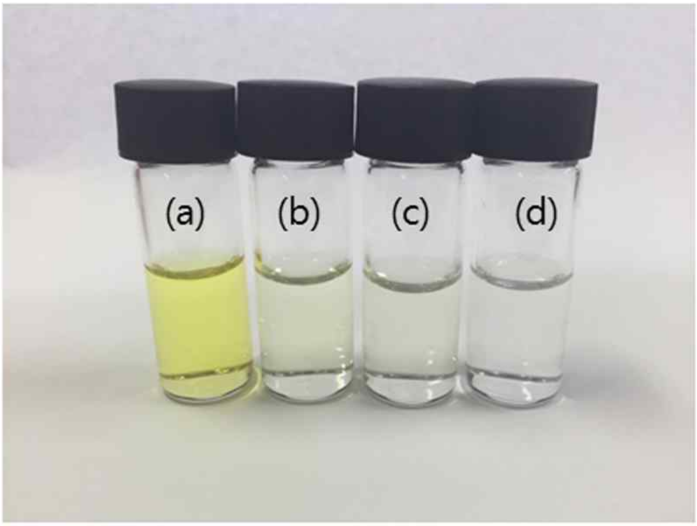

Fig. S4 High concentrated Au solution images (a) before and (b) after reaction. Low concentrated Au solution images (c) before and (d) after reaction.

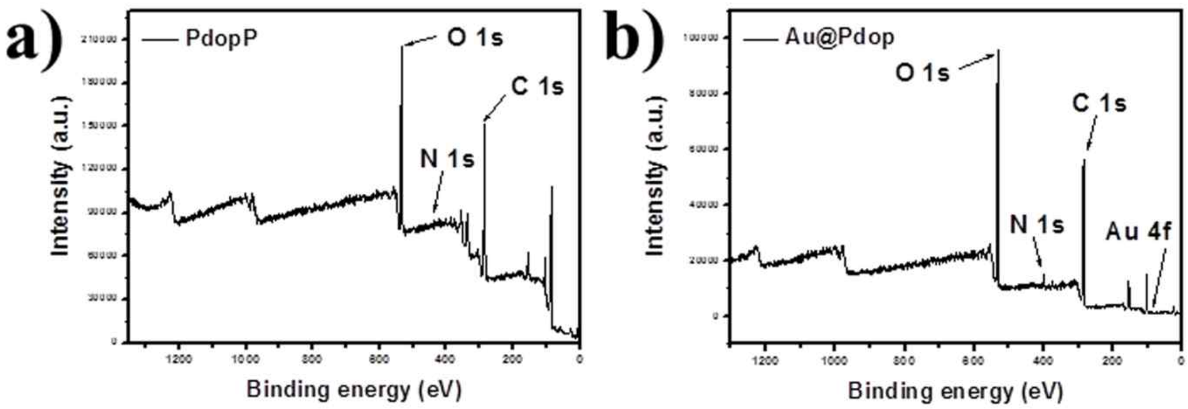

Fig. S5 XPS survey spectra of (a) PdopPs and (b) Au@Pdop NPs. XPS survey spectrum of the Au@Pdop NPs clearly shows the C 1s, N 1s, O 1s, and Au 4f peaks, which confirms the presence of Pdop shell on AuNPs.

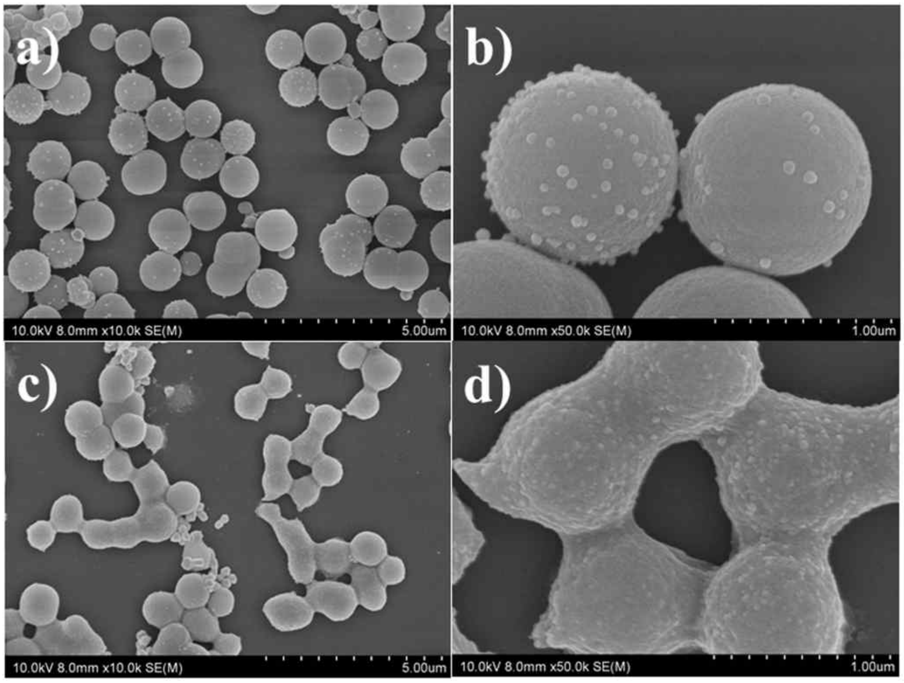

Fig. S6 SEM images of PdopP-Au_3 (a, b) before and (c, d) after release of Au@Pdop NPs for 120 hours.

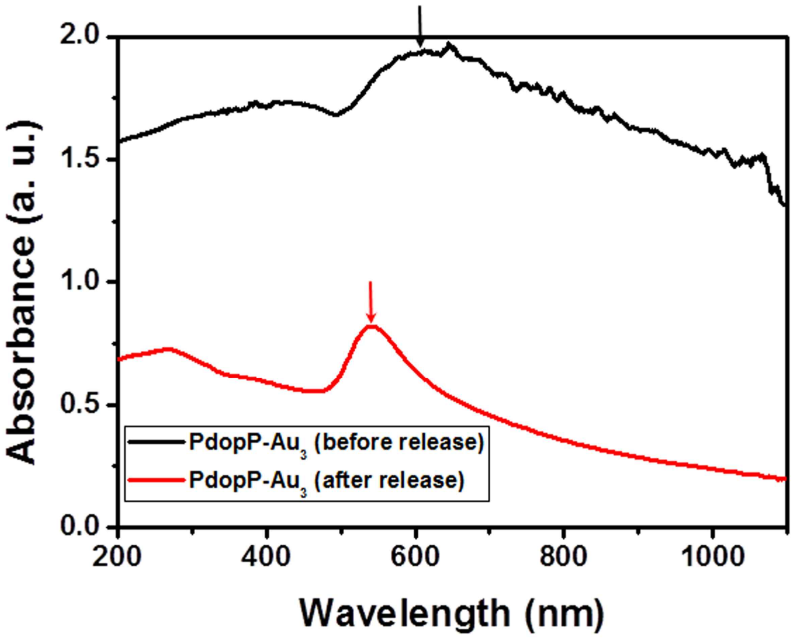

Fig. S7 UV-vis spectra of the PdopP-Au_3 (black line) before and (red line) after release of the Au@Pdop NPs. After release of NPs, the absorbance of the PdopP-Au_3 was decreased and the peak position of the PdopP-Au_3 was blue-shifted, compared to the PdopP-Au_3 before release.

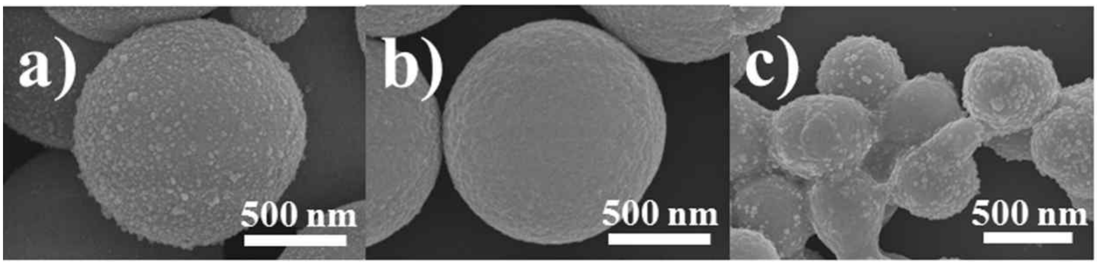

Fig. S8 SEM images of (a) PdopP-Ag_3, (b) PdopP-Pt_3, and (c) PdopP-Au_3 after release test.

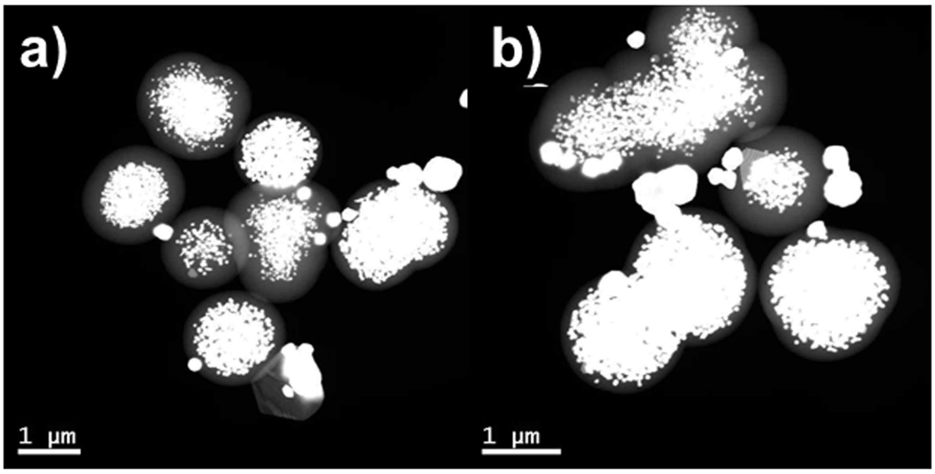

Fig. S9 STEM images of the PdopP-Au_3 (a) before and (b) after treatment of NaOH (pH=9.8). No significant change was observed.

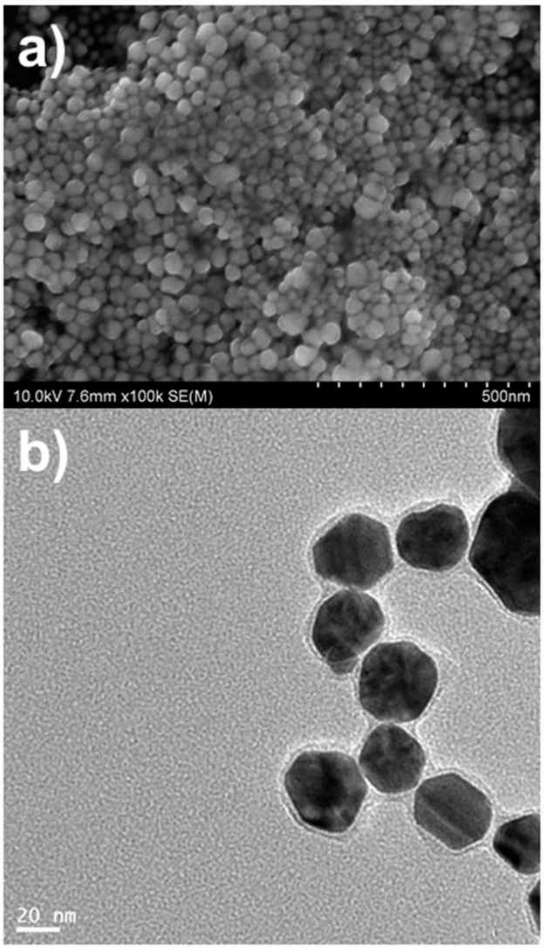

Fig. S10 (a) SEM and (b) TEM images of the Au@Pdop NPs released after 3 minutes of NIR irradiation on the PdopP-Au_3. The released NPs show core@shell structures.

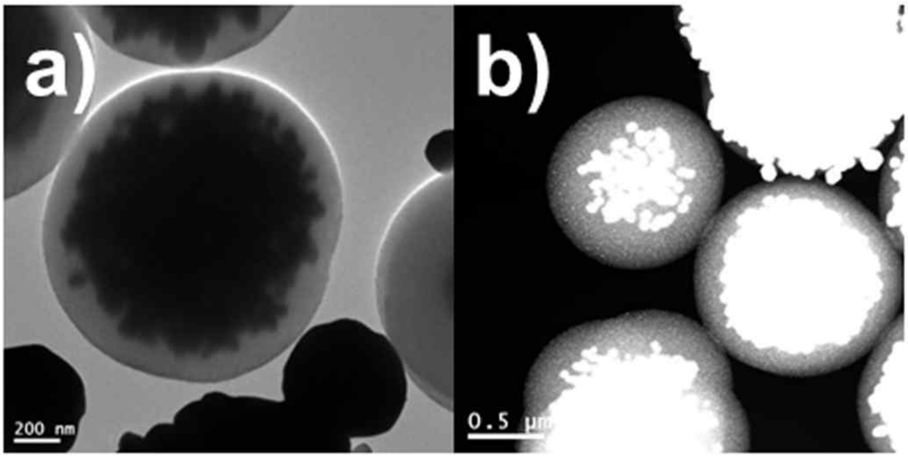

Fig. S11 (a) TEM and (b) STEM images of carbonized PdopP-Au_3 after release test. The carbonized PdopP-Au_3 was prepared by carbonization of PdopP-Au_3. No release of NPs was observed and no significant change in internal structures of carbonized PdopP-Au_3 was observed even under NIR irradiation.

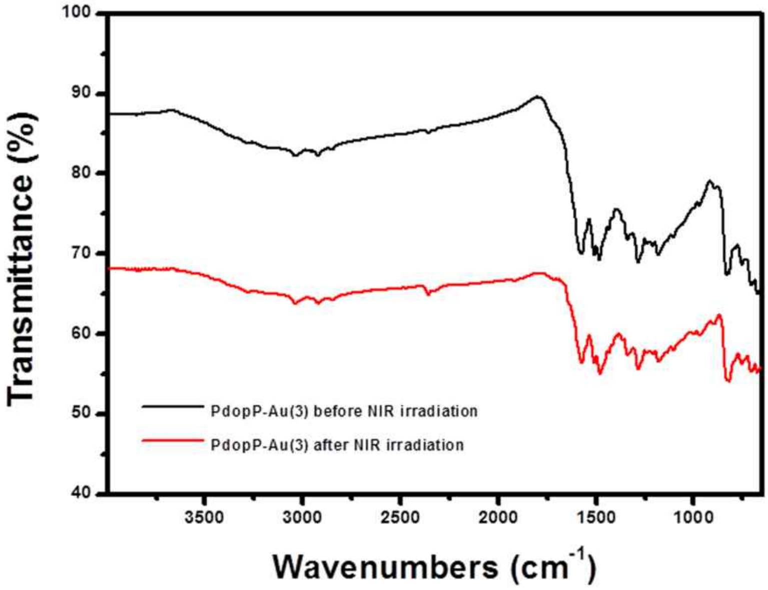

Fig. S12 FT-IR spectra of PdopP-Au_3 (black line) before and (red line) after NIR irradiation.

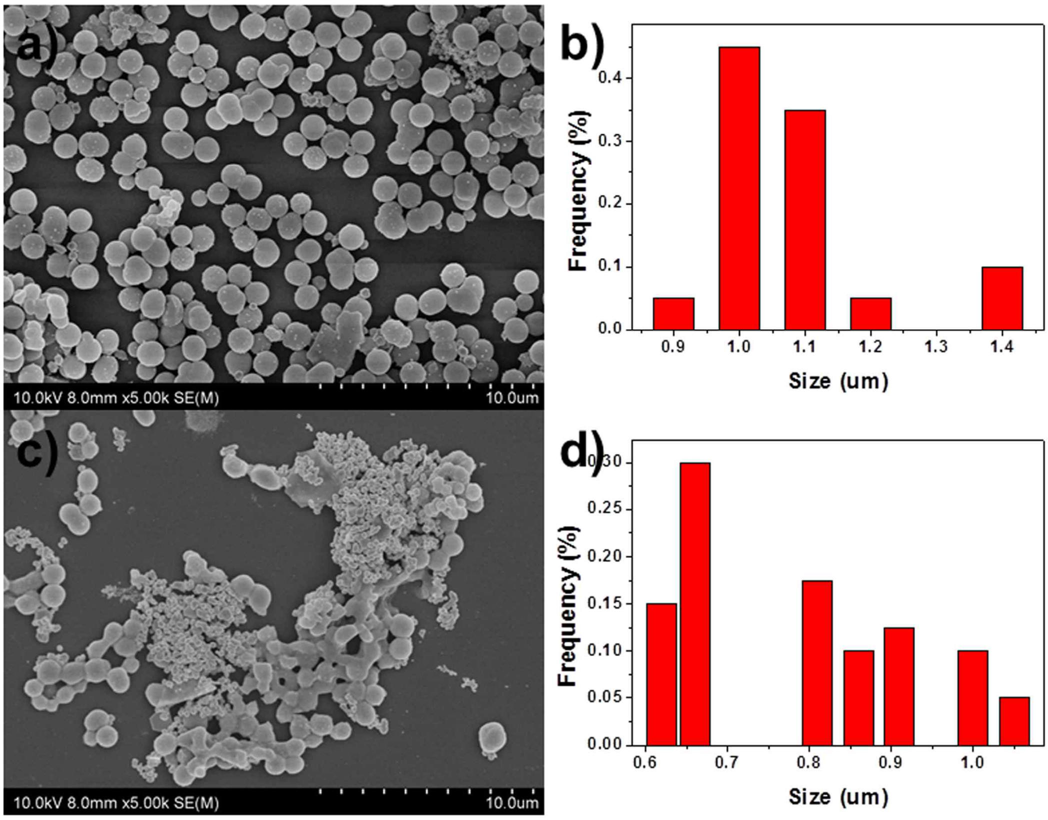

Fig. S13 SEM images and their corresponding average sizes of the PdopP-Au_3 (a, b) before and (c, d) after release of the Au@Pdop NPs stimulated by the NIR irradiation for 6 h.
